# Physiological, transcriptome and co-expression network analysis of chlorophyll-deficient mutants in flue-cured tobacco

**DOI:** 10.1186/s12870-023-04169-z

**Published:** 2023-03-22

**Authors:** Yuqing Feng, Yujing Li, Yuanyuan Zhao, Hongzhi Shi

**Affiliations:** College of Tobacco, Henan Agricultural University, Zhengzhou, 450000 Henan P. R. China

**Keywords:** Flue-cured tobacco, Chlorophyll deficient, Nitrate, RNA-sequencing, Co-expression network

## Abstract

**Background:**

Photosynthetic pigments in higher plants, including chlorophyll (Chl) and carotenoids, are crucial for photosynthesis and photoprotection. Chl-deficient tobacco seedlings generally have a lower photosynthesis rate and higher nitrate-nitrogen (NO_3_-N) content, which causes a profound influence on tobacco yield and quality. In this study, a stable albino leaf mutant (*Al*) and slight-green leaf mutant (*SG*) obtained from the common flue-cured tobacco (*Nicotiana tabacum L.*) cultivar ‘Zhongyan 100’ (ZY100) by mutagenesis with ethyl methanesulfonate (EMS) were used as materials. The differences between the Chl-deficient mutants and the wild-type (WT) were analyzed in terms of biomass, photosynthetic fluorescence parameters, and carbon- and nitrogen-related physiological parameters. RNA sequencing (RNA-seq) and weighted gene co-expression network analysis (WGCNA) were used to explore the key pathways and candidate genes regulating differentiated chlorophyll and nitrate content.

**Results:**

The results showed that, when compared to the WT, the Chl content and biomass of mutant plants were considerably lower while the NO_3_-N content was substantially elevated. The net photosynthetic rate, photosynthetic fluorescence parameters, carbohydrate, soluble protein, and carbon- and nitrogen-related enzyme activities all decreased in leaves of mutants and the development of chloroplasts was abnormal. Applying more nitrogen improved the growth and development of mutants, whereas NO_3_-N content distinctively increased compared with that of the WT. Through transcriptome sequencing, the downregulated genes in mutants were enriched in plant hormone signal transduction and nitrogen metabolism, which are involved in pigment biosynthesis and the carbon fixation pathway. In addition, two hub genes and seven transcription factors identified from the blue module through WGCNA were likely to be key candidate factors involved in chlorophyll synthesis and nitrate accumulation.

**Conclusion:**

Our results demonstrated that differences in chlorophyll and nitrate content were caused by the combined effects of chloroplast development, photosynthesis, as well as related biological activity. In addition, transcriptome results provide a bioinformatics resource for further functional identification of key pathways and genes responsible for differences in chlorophyll and nitrate content in tobacco plants.

**Supplementary Information:**

The online version contains supplementary material available at 10.1186/s12870-023-04169-z.

## Background

Photosynthesis provides substrate and energy for secondary metabolism in plants [1] and acts as one of the major determinants of carbon balance and growth in plants [2]. Besides, the photosynthates can be provided as the carbon skeleton for plant assembly, cell metabolism, material transportation, and nitrogen metabolism [3, 4]. Crop yield is heavily correlated with plant growth and over 90% of crop biomass is derived from photosynthetic products [5]. The ability and efficiency of photosynthesis are of great value for improving the productivity and quality of crops. Tobacco (*Nicotiana tabacum L.*) is a leaf-used plant and is considered to be an economically significant crop. Photosynthesis is crucial for the yield and quality of tobacco leaves. In addition, a reduced photosynthesis rate (Pn) leads to the accumulation of nitrate [3], which is the precursor of tobacco-specific nitrosamines (TSNAs). TSNAs are potent carcinogens that occur at high levels in tobacco products and can cause cancer in humans [6, 7]. Therefore, improving photosynthesis should be justified to decrease the risk of TSNA exposure to the portion of the population that uses tobacco products.

Photosynthetic pigments, including chlorophyll (Chl) and carotenoids, are involved in harvesting and converting light through photosynthesis into chemical energy that plants use to supply both carbon and other biochemical products for growth and development. Burley tobacco seedlings, which is homozygous for recessive mutant alleles at the *Yellow Burley 1* (*Yb*_1_) and *Yellow Burley 2* (*Yb*_2_) loci, had higher nitrate and TSNA accumulation [8]. Besides, the biomass of burley tobacco was significantly lower than that of flue-cured tobacco, and a similar leaf biomass was achieved when the burley tobacco was given six-fold more nitrogen fertilizers, but a higher nitrate content was observed [3]. We hypothesized that changes in some genes and pathways resulted in the lack of Chl, which in turn affected the photosynthesis efficiency and enzyme activities of plants and had adverse effects on the chemistry matrix, such as producing higher levels of nitrate and TSNA. However, the link between a lack of Chl and abnormal nitrate accumulation is still unclear, and the hypothesis remains to be verified. In this study, Chl-deficient tobacco seedlings were employed to determine the underlying mechanisms of chlorophyll biosynthesis and the relationship between chlorophyll and nitrate contents.

The entire Chl biosynthesis process starts from L-glutamine-tRNA and ultimately forms Chl a and Chl b through a multistep enzymatic reaction involving 15 enzymes and more than 20 genes [9, 10]. During this process, some transcriptional regulators also strongly affected the efficiency of chlorophyll synthesis and the development of chloroplasts, such as the LONG HYPOCOTYL 5 (HY5) protein [11], multiple organellar RNA editing factors (MORFs) [12], and the NAC transcription factor (TF) [13], WRKY [14], and MYB families [15]. Additionally, studies have reported that the expression of some genes related to chloroplasts also affect their biogenesis. The resultant disruption in Chl metabolism and chloroplast assembly can lead to abnormal leaf color [16, 17].

Many yellow-green leaf color mutants in plants, including wheat [18], rice [19], apple [20], Chinese cabbage [21], and tea [22] have been discovered. Also, methylome, metabolomics, and transcriptomics technical methods have been employed to explore the potential mechanisms underlying leaf color variation. However, the physiological and molecular mechanism of Chl biosynthesis and nitrate accumulation in Chl-deficient tobacco seedlings remains unclear. In this study, Chl-deficient mutants were used to explore the underlying molecular mechanisms in response to Chl deficiency in tobacco. Meanwhile, the changes of Chl-deficient tobacco in response to low and high nitrogen levels were also investigated. Specifically, we sought to identify key differentially expressed genes (DEGs) underlying the molecular mechanisms of chl differentiation and nitrate accumulation and further identify the relationship between Chl and nitrate content of Chl-deficient tobacco. Our results provide a theoretical basis for further investigating the mechanism of Chl metabolism and in response to Chl deficiency, thus laying a solid foundation for the improvement of nitrogen-use efficiency and risk reduction in tobacco products via genetic engineering in the future.

## Results

### Analysis of morphological characteristics and chloroplast ultrastructure

**Fig. 1:**
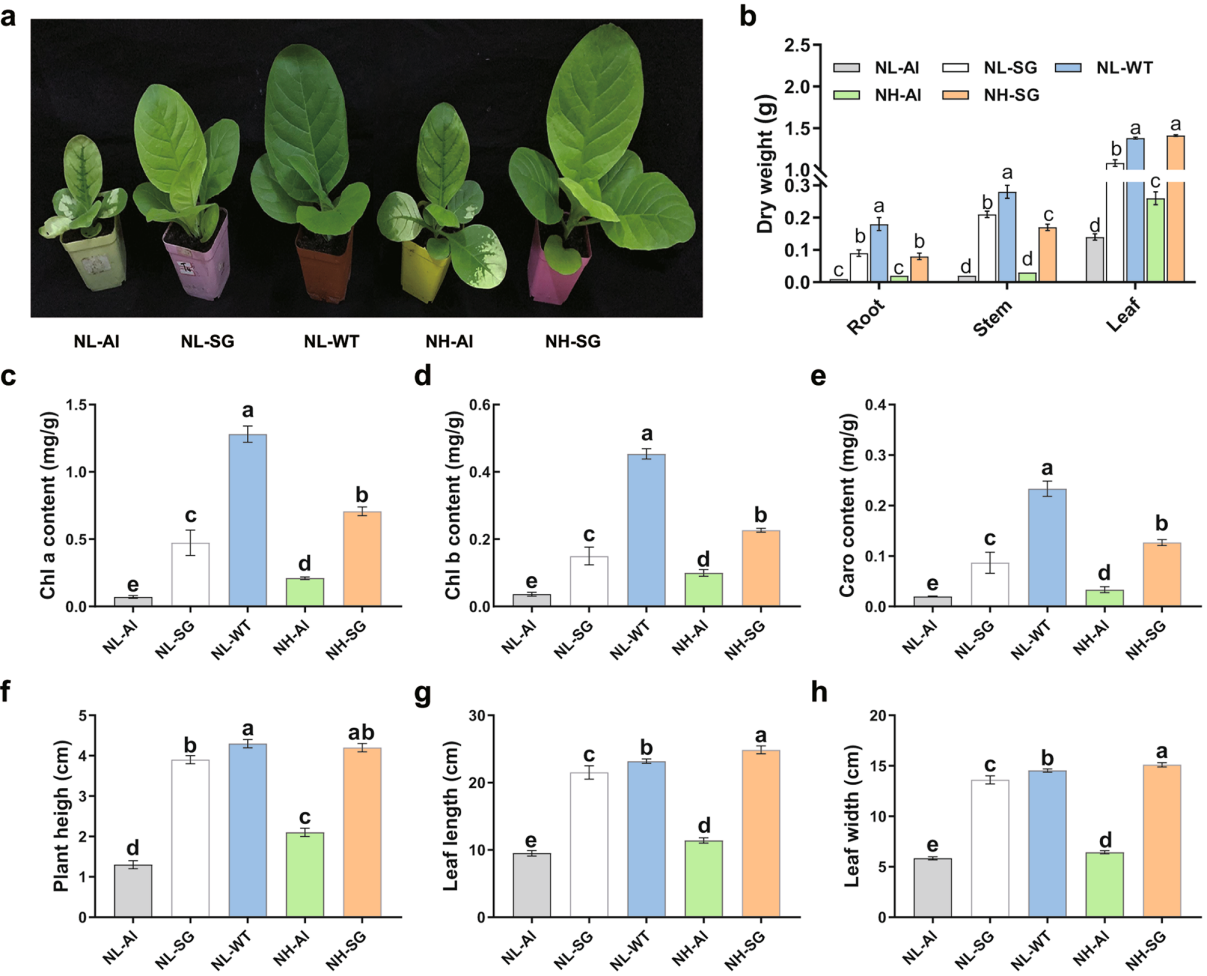
Analysis of morphological characteristics. (**a**) The phenotype of Chl-deficient and WT tobacco seedlings. (**b**) The dry weight of root, stem, and leaf. (**c**) Chl a content. (**d**) Chl b content. (**e**) Carotenoid. (**f**) Plant height. (**g**) Leaf length. (**h**) Leaf width. Error bars indicate the standard error of the mean (n = 3 individuals per treatment group). Small letters indicate significant differences between treatments at *p <* 0.05

The phenotype, pigment content, and biomass accumulation are displayed (Fig. 1a-h). The results showed that the growth of Al and SG mutants was inhibited at 5 mM nitrogen application (Fig. 1a). The root and stem dry weight of Al and SG mutants at both 5 mM and 20 mM nitrogen application was lower than that of WT. Additionally, the dry weight of SG leaves at 20 mM was similar to the WT at 5 mM (Fig. 1b). Furthermore, the Chl a, Chl b, and carotenoid contents of Al and SG mutants at both 5 mM and 20 mM were significantly lower than those of WT. The Chl a, Chl b, and carotenoid contents of the mutants were lower by 94.53%, 91.11%, and 91.30%, respectively, in Al leaves in comparison to WT leaves at 5 mM nitrogen application. Additionally, the Chl a, Chl b, and carotenoid contents were lower by 63.28%, 66.67%, and 60.87%, respectively, in SG leaves relative to WT leaves at 5 mM nitrogen application (Fig. 1c–e). The plant height, leaf length, and leaf width of Al and SG mutants at 5 mM were significantly lower than those of the WT (Fig. 1f–h).

**Fig. 2:**
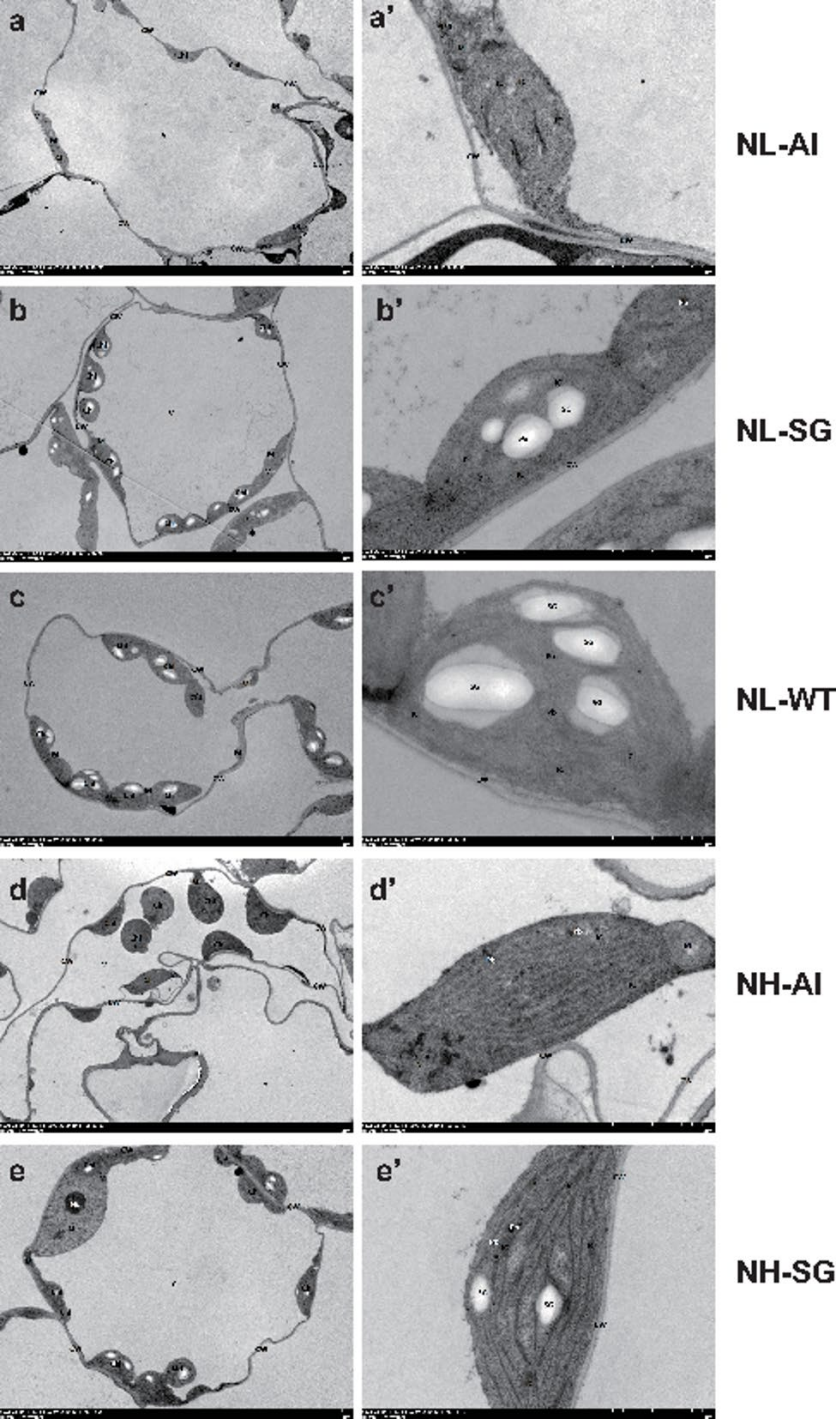
Chloroplast ultrastructure of Chl-deficient mutants and WT tobacco seedlings. The right panels (a–e) represent higher magnification than the left panels. (**a–e**), Chloroplast ultrastructure of NL-Al, NL-SG, NL-WT, NH-Al, NH-SG. CW, Cell wall; Chl, Chloroplast; SG, starch granule; IC, internal capsule; Pb, Plastoglobules; M, mitochondria; RER, Rough endoplasmic reticula; V, *V* vesicle-like structure

The ultrastructure of the chloroplasts was observed by electron microscope. Under 5 mM nitrogen application conditions, the cell structure was disorganized, the number of chloroplasts and plastoglobules (Pb) in Al decreased, and no starch grains were found in the chloroplasts. The structure of SG was normal, while the area of chloroplasts and starch grains were obviously lower than that of WT. Under 20 mM nitrogen application conditions, the structure of Al was also disorganized. Additionally, the chloroplast and starch grain areas of SG were slightly increased, but still lower than that of WT tobacco (Fig. 2).

### Assessment of enzyme activities, photosynthetic parameters, and carbonitride content

**Fig. 3:**
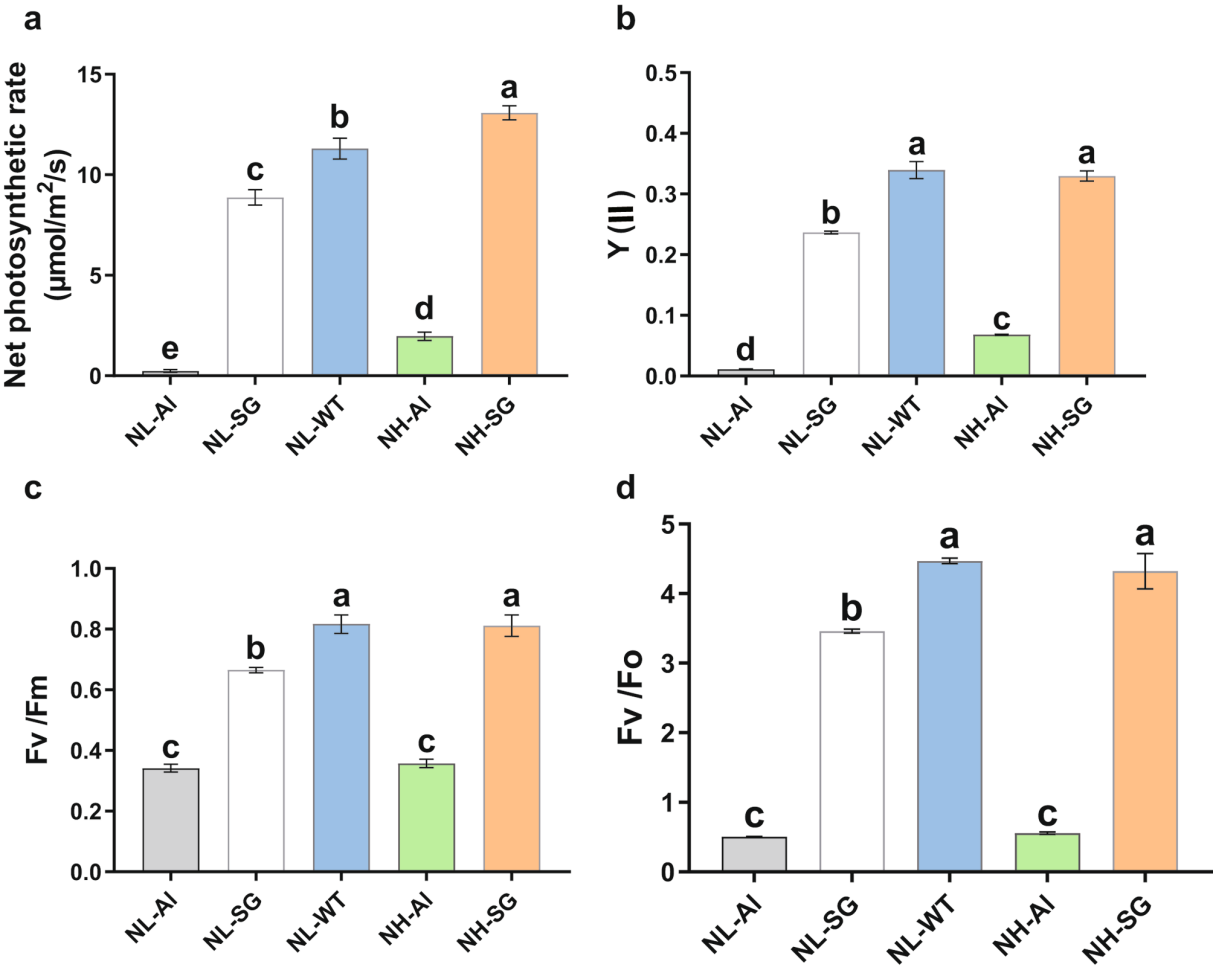
Differences in net photosynthesis and chlorophyll fluorescence parameters of tobacco leaves. (**a**) Net photosynthetic rate. (**b**) Y(II). (**c**) Fv/Fm. (d) Fv/Fo. Y(II), the actual photochemical efficiency of PSII (Y(II)); Fv/Fm, PSII maximum photochemical efficiency; Fv/Fo, and potential photochemical activity (Fv/Fo). Error bars indicate the standard error of the mean (n = 3 individuals per treatment group). Small letters indicate significant differences between treatments at *p <* 0.05, respectively

In order to investigate the effects of Chl-deficiency in Al and SG mutants, we measured the photosynthetic parameters and enzyme activities. The Pn in Al and SG was significantly lower in comparison to WT at 5 mM nitrogen application while increasing the nitrogen application enhanced the Pn in Chl-deficient seedlings (Fig. 3a). Chl fluorescence induction experiments demonstrated that the actual photochemical efficiency of PSII (Y(II)), PSII maximum photochemical efficiency (Fv/Fm), and potential photochemical activity (Fv/Fo) were also significantly higher in WT than Al and SG mutants at 5 mM nitrogen application (Fig. 3b–d). These results indicated that WT tobacco leaves had a greater photosynthetic capacity than its Chl-deficient mutants. Additionally, an increased amount of nitrogen fertilizer application generally gave rise to higher photosynthetic capacity in Chl-deficient mutants. Furthermore, it was noted that no significant difference was observed in Chl fluorescence parameters between the SG at 20 mM nitrogen application and WT at 5 mM nitrogen application, suggesting that the photosynthetic capacity in Chl-deficient mutants could be restored to normal levels by high-level nitrogen application.

**Fig. 4:**
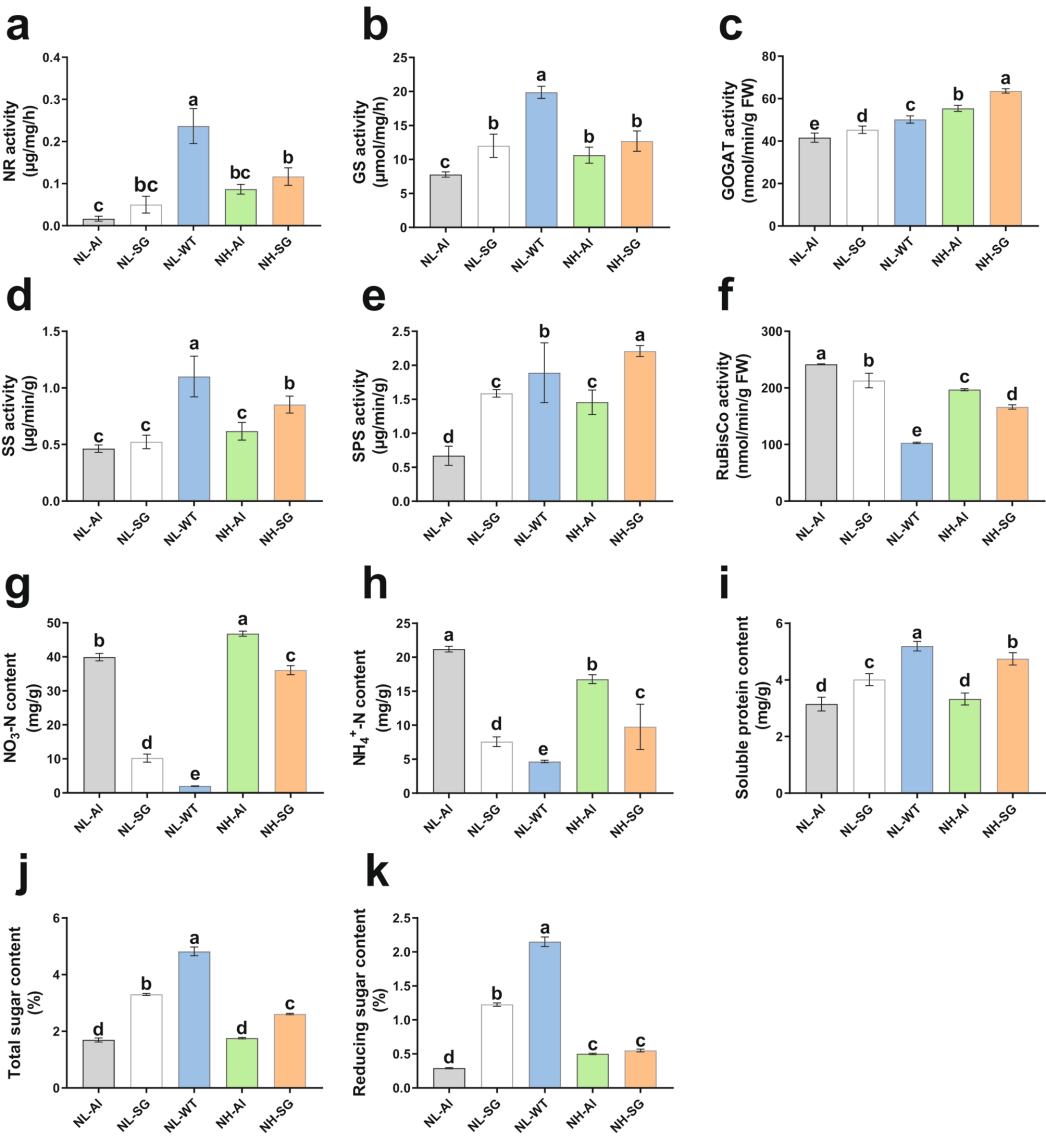
Assessment of enzyme activities and carbonitride content. (**a**) NRA. (**b**) GSA. (**c**) GOGAT. (**d**) SSA. (**e**) SPS activity. (**f**) Rubisco activity. (**g**) NO_3_-N content. (**h**) NH_4_-N content. (**i**) Soluble protein content. (**j**) Total sugar content. (**k**) Reducing sugar content. Error bars indicate the standard error of the mean (n = 3 individuals per treatment group). Small letters indicate significant differences between treatments at *p <* 0.05, respectively

The sucrose synthetase activity (SSA), sucrose phosphate synthase (SPS), rubisco enzyme activity, nitrate reductase activity (NRA), glutamine synthetase activity (GSA), and glutamate synthase (GOGAT) activity in the leaves of the NL-Al and NL-SG were lower than that of the NL-WT while these enzyme activities in NL-Al and NL-SG leaves increased with the increase of nitrogen application (Fig. 4a–e). On the contrary, the rubisco activity in the leaves of the Al and SG was substantially higher relative to the WT, which was a compensating reaction in response to the Chl deficiency (Fig. 4f). Moreover, a significantly higher NO_3_-N content was observed in Chl-deficient mutants in comparison to WT leaves at 5 mM nitrogen application (18.85 times for Al and 4.09 times for SG). Notably, the NO_3_-N content in SG was increased by 16.96 times compared with the WT when they had similar leaf biomasses (Fig. 4g). Finally, the soluble protein, total sugar, and reducing sugar contents were all lower in the NL-Al and NL-SG leaves than those of NL-WT while the NH_4_-N content was higher than that of NL-WT (Fig. 4h–k).

### Sequencing data results

15 cDNA libraries were established for the RNA-seq analysis. A total of 123.81, 120.12, 119.33, 115.73, and 141.42 million raw reads were identified in the NL-Al, NL-SG, NL-WT, NH-Al, and NH-SG groups, respectively. After data processing, 117.07 (94.56%), 113.20 (94.24%), 111.19 (93.18%), 109.63 (94.73%), and 133.31 (94.27%) million clean reads were obtained for further analysis (Table S1). Over 92% of the clean reads across all samples were mapped to the tobacco reference genome. Over 90% of the sequencing data showed quality ≥ 30 (Q30), implying successful library construction and reliability for subsequent bioinformatics analysis.

### Analysis of the DEGs between mutants and wild-type

**Fig. 5:**
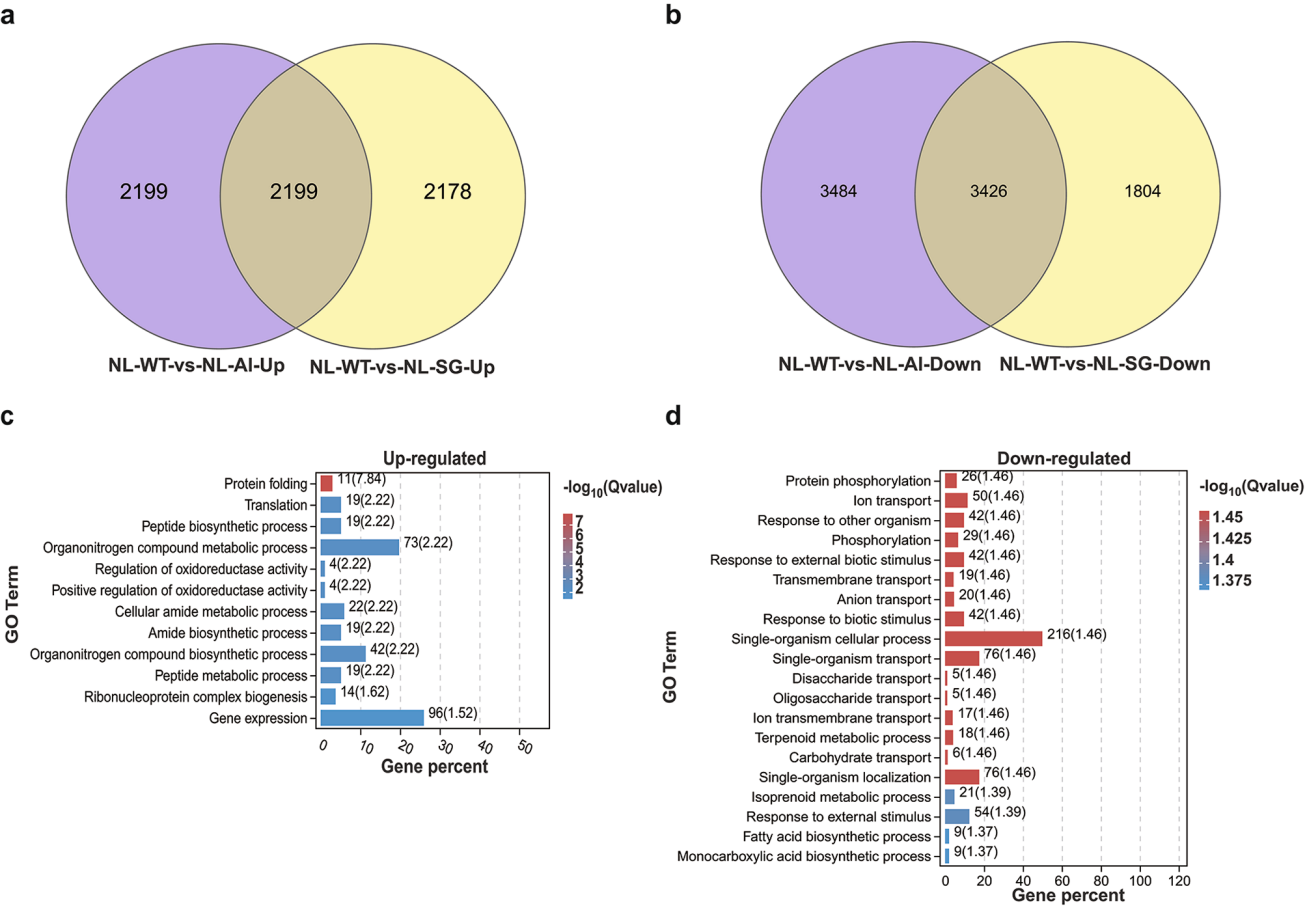
Identification of the DEGs between mutant and wild-type and Gene Ontology (GO) analysis. (**a**) Venn diagrams of upregulated DEGs. (**b**) Venn diagrams of downregulated DEGs. Top 20 of KEGG pathway analysis of DEGs. (**c**) GO enrichment results of upregulated DEGs. (**d**) GO enrichment results of downregulated DEGs. GO terms with FDR < 0.05 are shown

A Venn diagram analysis between NL-WT_vs_NL-Al and NL-WT_vs_NL-SG groups of DEGs was performed. 2199 upregulated and 3426 downregulated DEGs were achieved (Fig. 5a,b). We further performed gene enrichment analysis on the genes and found that the GO terms in upregulated genes were mainly enriched in protein folding, translation, peptide biosynthetic process, organonitrogen compound metabolism process, and regulation of oxidoreductase activity (Fig. 5c); and the GO terms in downregulated genes were mainly enriched in protein phosphorylation, ion transport, response to other organisms, phosphorylation, and response to external biotic stimulus (Fig. 5d).

**Fig. 6:**
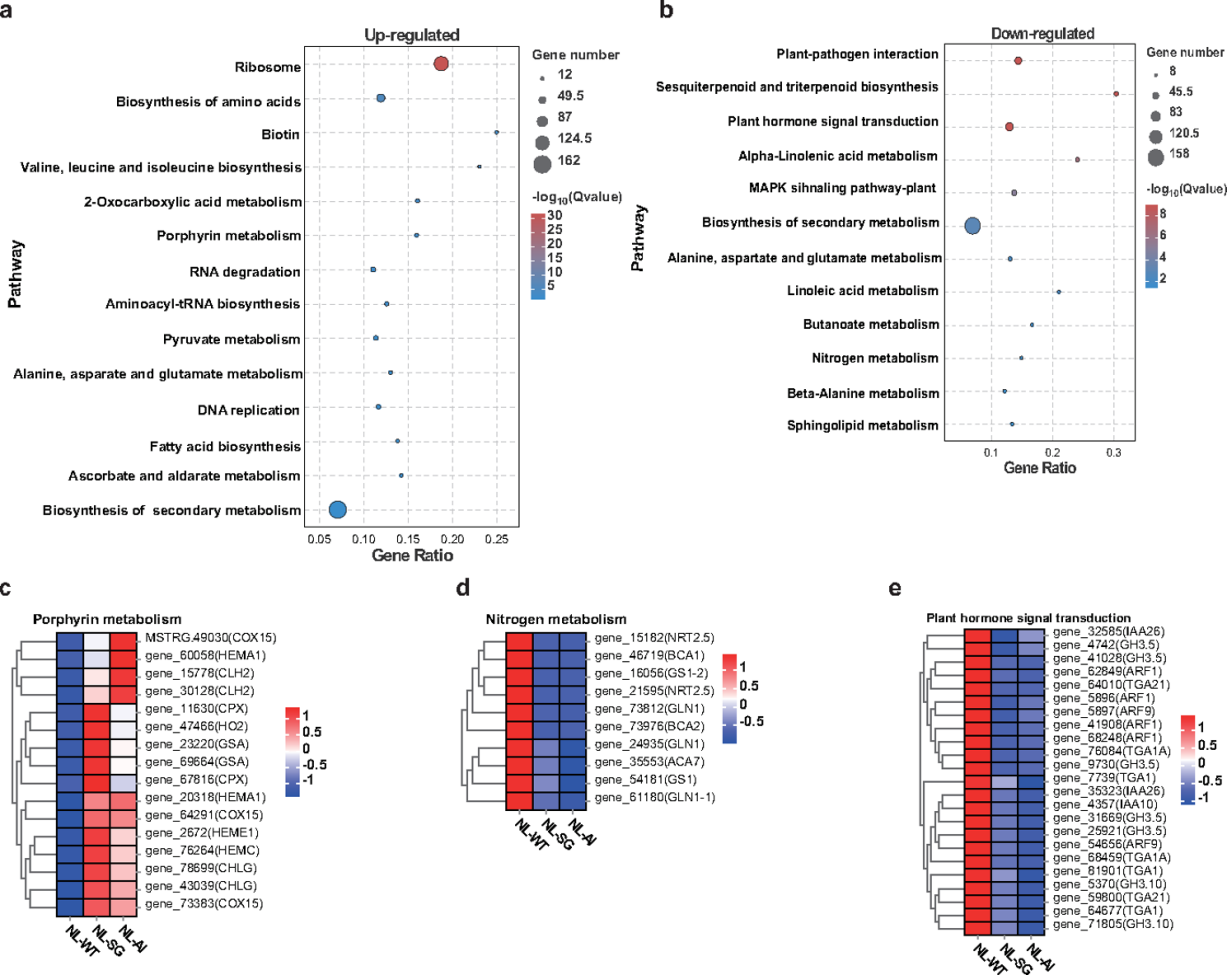
KEGG analysis and key gene expression pattern. (**a**,**b**) KEGG pathway analysis of upregulated and downregulated DEGs. Pathways with FDR < 0.05 are shown. (**c**) Heatmap of the key regulatory genes involved in porphyrin metabolism. (**d**) Heatmap of the key regulatory genes involved in nitrogen metabolism. (**e**) Heatmap of the key regulatory genes involved in plant hormone signal transduction

To obtain a functional insight into the DEGs, the upregulated and downregulated DEGs KEGG pathway analysis (Fig. 6a, b) were performed. We found “ribosome” and “porphyrin metabolism” were enriched in upregulated DEGs. The downregulated DEGs were enriched in some key metabolic pathways, such as “plant hormone signal transduction” and “nitrogen metabolism”. The genes involved in porphyrin metabolism showed higher levels of expression in mutants compared to the wild-type (Fig. 6c), which was a compensating reaction in response to the Chl deficiency. By contrast, the key genes correlated with nitrogen metabolism showed opposite expression trends (Fig. 6d) and most auxin response genes were also downregulated in mutant plants, such as *gene_5896* (*ARF1*), *gene_5897* (*ARF9*), and *gene_32585* (*IAA26*) (Fig. 6e), which were the possible factors related to the inhibition of growth of seedlings with albino and slight-green leaf mutations.

**Fig. 7:**
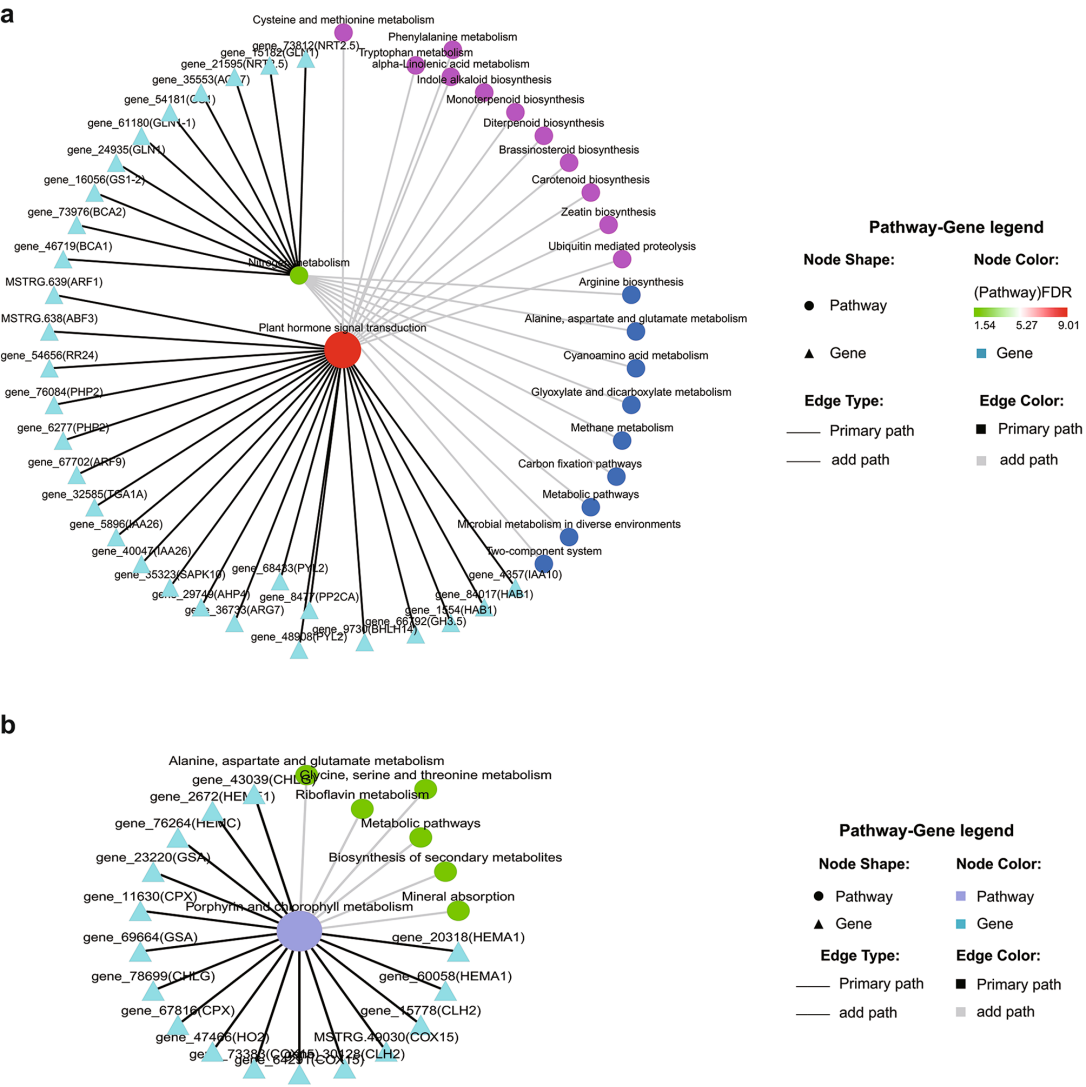
KEGG network of the key pathways in upregulated and downregulated DEGs between mutant and wild-type tobacco plants. (**a**) KEGG network of the key pathways in downregulated DEGs. (**b**) KEGG network of the key pathway in upregulated DEGs. The circular nodes in different colors indicate KEGG pathways. Circular node size represents the number of genes enriched in this pathway. The nodes in the sky-blue triangle represent genes. The solid lines indicate connections between two or more pathways or between pathways and genes. The gray lines represent adjacent pathways supplemented from the KEGG connection database

Interestingly, the KEGG network indicated that the plant hormone signal transduction pathway has a connection with carotenoid biosynthesis, zeatin biosynthesis, ubiquitin mediated proteolysis, diterpenoid biosynthesis, monoterpenoid biosynthesis, tryptophan metabolism, and brassinosteroid biosynthesis (Fig. 7a), which have a direct or indirect influence on pigment content and plant growth. Meanwile, the nitrogen metabolism has a connection with alanine, aspartate and glutamate metabolism, cyanoamino acid metabolism, arginine metabolism, and carbon fixation pathways (Fig. 7a). Furthermore, the porphyrin and chlorophyll metabolism was connected to alanine, aspartate and glutamate metabolism, glycine, serine and threonine metabolism, biosynthesis of secondary metabolites, and mineral absorption, indicating that amino acid metabolism and secondary metabolism exert an influence on pigment content (Fig. 7b).

### Trend analysis of DEGs enrichment of mutant tobacco seedlings under different nitrogen levels

**Fig. 8:**
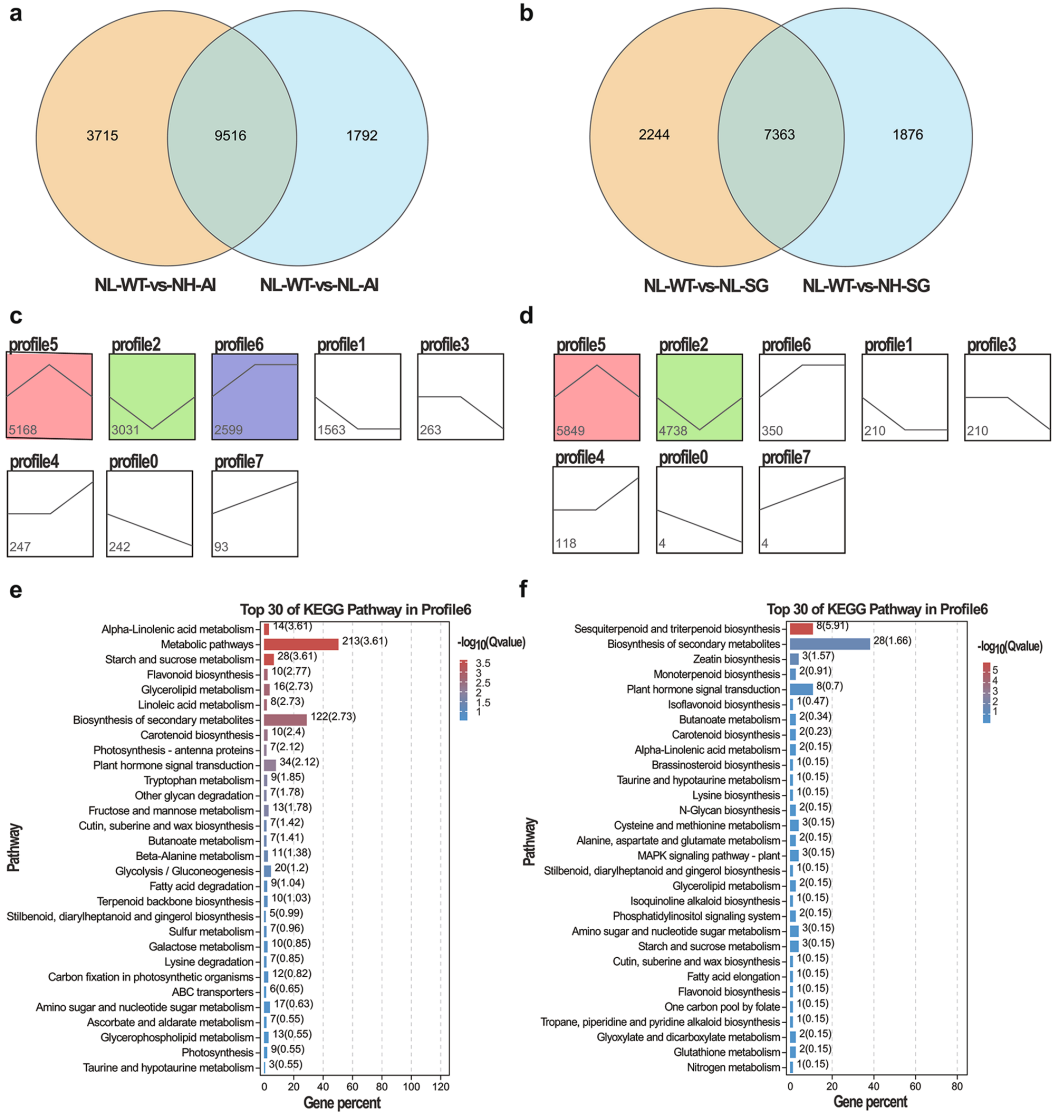
Trend analysis of DEGs enrichment under different nitrogen levels. (**a**,**b**) Venn diagram analysis. (**c**,**d**) Trend analysis of differentially expressed genes. The 15,023 DEGs from NL-Al, NL-WT and NH-Al were clustered into 8 profiles. The 11,483 DEGs from NL-SG, NL-WT and NH-SG were clustered into 8 profiles. (**e**,**f**) Top 30 of KEGG pathway enrichment of DEGs in Profile 6

The 15,023 DEGs from NL-Al, NL-WT, and NH-Al and 11,483 DEGs from NL-SG, NL-WT, and NH-SG were all clustered into 8 profiles (Fig. 8a,b). In Profile 6, the gene expression levels of Al and SG were downregulated in WT while they were upregulated after high-level nitrogen application. Therefore, we focused on the gene expression in Profile 6. KEGG pathway analysis showed DEGs in Profile 6 were all mapped in plant hormone signal transduction, carotenoid biosynthesis, starch and sucrose metabolism, and nitrogen metabolism (Fig. 8c,d), indicating the key pathways in response to nitrogen application in tobacco plants.

### WGCNA identifies candidate modules associated with pigment and NO_3_-N contents

**Fig. 9:**
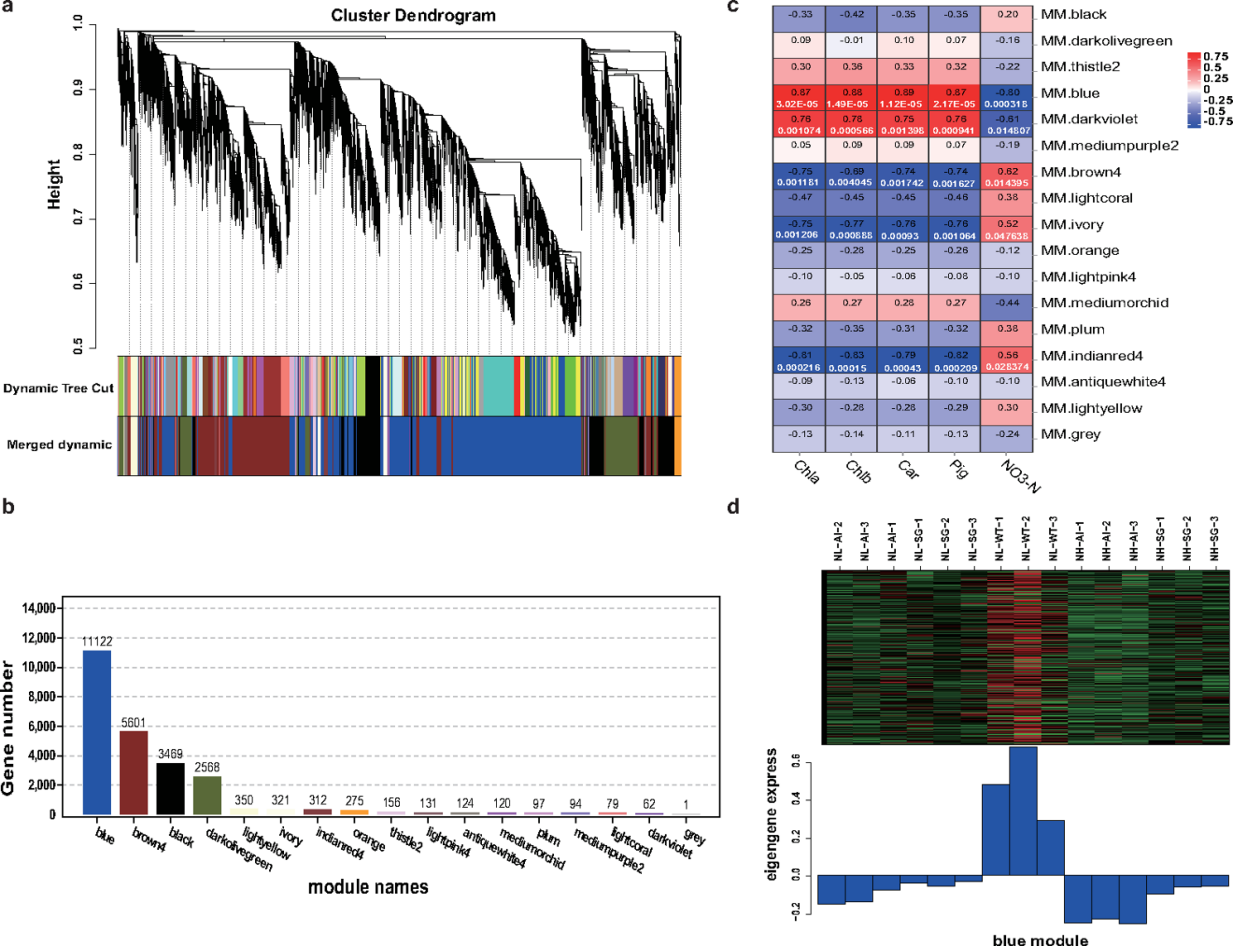
WGCNA and module-trait correlation analysis. (**a**) Hierarchical cluster tree showing co-expression modules identified by the Dynamic Tree Cut method. Each leaf, that is a short vertical line, corresponds to a gene. Branches of the dendrogram group together densely interconnected constitute modules and are labeled with different colors. Genes with highly co-expression levels (correlation ≥ 0. 75) were merged into one module, 17 modules were obtained in total. (**b**) The number of genes in each module. (**c**) Correlations of pigment and NO_3_-N contents with WGCNA modules. Each row corresponds to a module. The columns correspond to phenotype traits. The color of each cell indicates the correlation coefficient between the module and traits. Red represents positive correlation and blue represents negative correlation. (**d**) The heatmap and bar plot with blue module eigengene

Genes involved in the regulation of the pigment and NO_3_-N traits were grouped into modules. All 24,882 genes were grouped into 17 modules according to the similarity of expression patterns (Fig. 9a). To find phenotype-specific modules, the correlation value between the expression patterns of each module and the pigment and NO_3_-N contents were measured. Meanwhile, the correlation value no less than 0.8 was used as a threshold to identify the key modules. Lastly, the blue module was identified as a key module and the gene number was 11,122 (Fig. 9b). A positive correlation was observed between the blue module and pigment parameters, while it had a negative correlation with NO_3_-N content (Fig. 9c). Moreover, the genes involved in the blue module were highly upregulated in NL-WT plants according to the heatmap and bar plot with module eigengene (Fig. 9d). This phenomenon may denote possible correlations between genes that determine different phenotype traits.

### Function enrichment analysis of genes and network visualizing in blue module

**Fig. 10:**
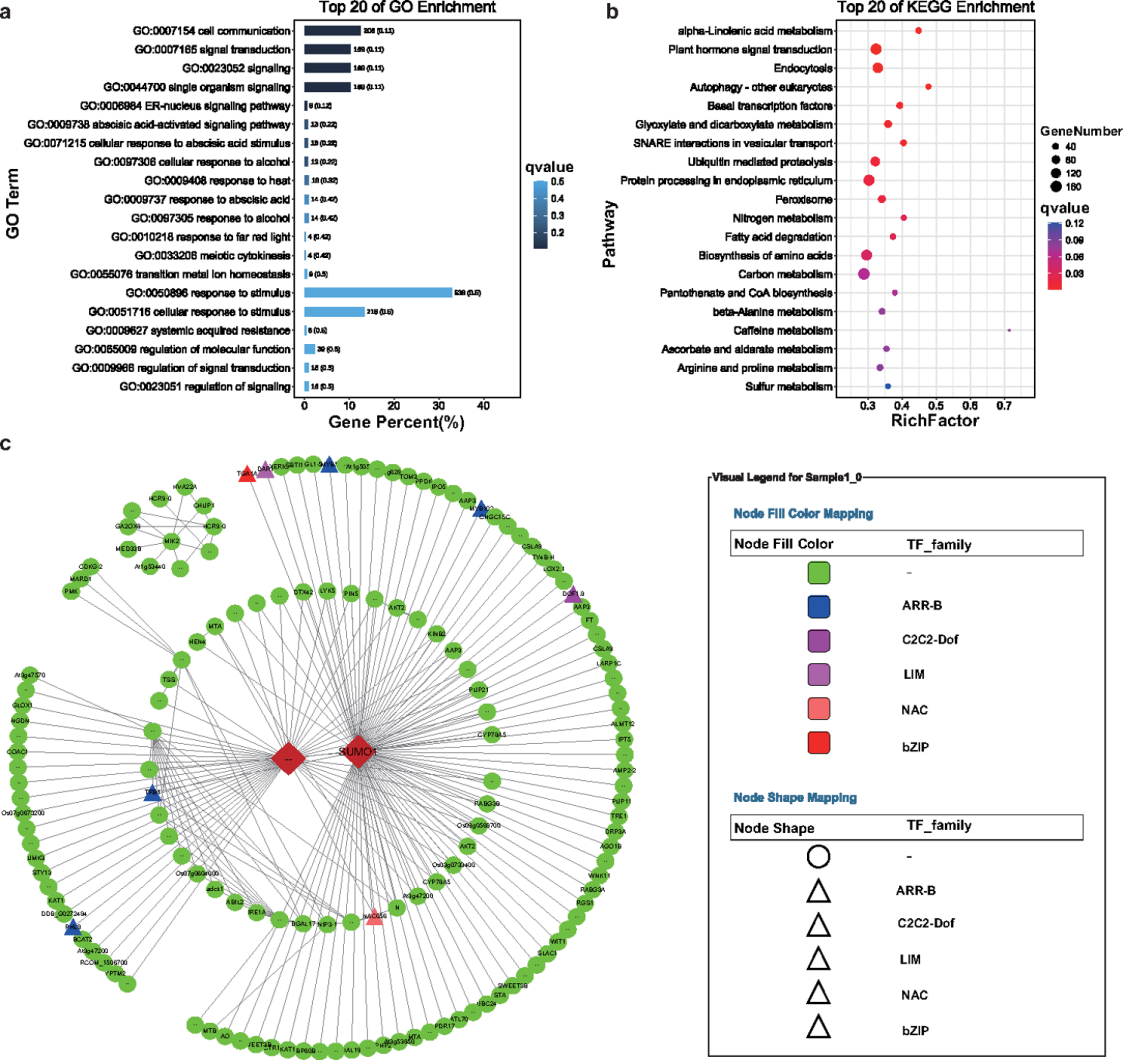
Function enrichment analysis of genes and co-expression network construction in blue module. (**a**) GO analysis of blue module; (**b**) KEGG analysis of blue module; (**c**) Co-expression network analysis of blue module

To reveal the specific functions played by each co-expressed module, a further GO and KEGG analysis of genes from the blue module was conducted. In the blue module, which was positively correlated with pigment content while negatively correlated with NO_3_-N content, GO analysis indicated that “cell communication” and “signal transduction” were the most significantly enriched biological processes (Fig. 10a). KEGG analysis indicated “alpha-Linolenic acid metabolism” and “plant hormone signal transduction” as the most significantly enriched metabolic pathways (Fig. 10b). Additionally, “nitrogen metabolism”, “biosynthesis of amino acids” and “carbon metabolism” pathways were enriched (Fig. 10b).

To further find key genes from the blue module, the top 200 edges ranked with weighted values were screened for co-expression network construction (Table S2). In the network, two hub genes were identified by CytoHubba plug-in using MCC algorithm in Cytoscape software (Fig. 5B). And based on gene annotation, the DETFs (differential expressed transfactors) co-expressed with the key genes were further determined (Table S3). The network diagram showed *TGA1A* (*gene_68459*), *MYB5* (*gene_32175*), *DAR1*(*gene_76554*), *MYB102* (*gene_51745*), *DOF1.8* (*gene_17063*), *NAC056* (*gene_18678*), and *TRB1* (*gene_14307*) were correlated with *SUMO1* (*MSTRG.12,427*) (Fig. 10c). In addition, the amino acid transporter *AAP3* and the sugar transporter *SWEET3B* were found to have a regulatory relationship with *SUMO1*.

### Validation of DEGs by quantitative real-time PCR (qRT-PCR)

In order to further verify the reliability of the transcriptome data, six DEGs were selected for qRT-PCR (Figure S1). The expression patterns of six genes detected by qRT-PCR were consistent with the transcriptome expression trend. The results showed that the transcriptome data was valid and reliable.

## Discussion

As an leaf-harvested cash crop, nitrate is a negative factor affecting flue-cured tobacco quality which contributes to the accumulation of TSNAs. The burley tobacco, which is a chloroplast-deficient mutant, is known to contain higher TSNA and nitrate concentrations than flue-cured tobacco [3, 23]. Previous studies have showed that the lack of chlorophyll affects the photosynthesis efficiency, thus leading to higher levels of nitrate [3]. But the linkage between chlorophyll lackness and nitrate accumulation in flue-cured tobacco is still unclear and the hypothesis remains to be verified. In our study, the chlorophyll content in tobacco leaves of Al and SG decreased substantially compared to WT (Fig. 1c–e), and the photosynthetic fluorescence-related indices (Pn, Fv/Fm, Fv/F0, Y(II), total sugar, and reducing sugar content) (Figs. 3 and 4j and k) all decreased distinctively under the same nitrogen level. The results were in line with previous studies reported by Shao et al. [24] and Lu et al. [18], indicating that reduced Chl content indeed has a profound influence on tobacco photosynthesis. Additionally, lower enzyme activities and higher NO_3_-N content were achieved compared to WT (Fig. 4a–i). Based on the downregulated DEGs between mutants and wild-type, we found that the sesquiterpenoid and triterpenoid biosynthesis, plant hormone signal transduction and nitrogen metabolism pathways were enriched. And the KEGG network analysis revealed that the nitrogen metabolism has a connection with alanine, aspartate and glutamate metabolism, cyanoamino acid metabolism, arginine metabolism, and carbon fixation pathways (Fig. 7a). A lower expression level of genes involved in these pathways would lead to reduced pigment, which in turn affects related protein synthesis and enzyme activities, leading to the accumulation of nitrate. Higher nitrate accumulation would therefore increase the risk of exposure to TSNA and reduce nitrogen use efficiency.

Chloroplasts are important organelles for photosynthesis in plants, and are also the site of synthesis and accumulation of photosynthetic pigments such as Chl and carotenoids [25]. In addition, the chloroplast ultrastructure results showed that variegated leaf color mutants exhibited varying degrees of structural change. A previous study [26] reported that the number of chloroplasts in the the yellow green peel (*ygp*) mutant of cucumber (*Cucumis sativus* L.) was lower than that in the wild-type. Similar structural changes were also detected in a study of leaf color mutants of tomatoes [27]. In this study, the cell structure was disorganized in albino mutants and no starch grains were found in chloroplasts. Although the cell structure of SG mutants was normal, it exhibited smaller chloroplast along with lower amount of starch grains than those of the WT (Fig. 2). The instability of chloroplast structure may cause chlorophyll flow into the cytoplasm, which is then destroyed by redox which may cause the yellow phenotype of plants [27]. Ribosomal proteins play essential parts in the translation of key proteins involved in chloroplast development and repair [28]. Transcriptome analysis uncovered that the upregulated genes between Chl-deficient mutants and the WT were most significantly enriched in ribosome (ko03010), which would affect Chl synthesis (Fig. 6a). The biosynthesis of Chl is regulated by many genes and any mutation would affect Chl accumulation [29]. In contrast, our study found the expression of *HEMA1* (*gene_20318*), *HEMC* (*gene_76264*), and *CHLG* (*gene_43039*, and *gene_78699*), encoding the enzymes for the Chl biosynthesis pathway, were all upregulated in mutant plants, which might be a compensating reaction in response to the abnormal chloroplast structure (Fig. 6c).

It is generally accepted that Chl plays an essential role in photosynthesis. The crop yield depends on Chl content and net photosynthetic rate. A previous study revealed that burley tobacco, a Chl-deficient mutant, had a lower leaf biomass and net photosynthetic rate compared with flue-cured tobacco [3]. Nitrogen is an important component of protein, nucleic acid, Chl, and some hormones in plants and rational nitrogen is conducive to the photosynthetic capacity and development of plants [17, 30]. Noor et al. reported that increasing nitrogen application can improve the Chl fluorescence parameters and photosynthetic pigment content of wheat [31]. In our study, the photosynthetic parameters, key enzyme activities related to carbon and nitrogen metabolism, were all improved by applying three times more nitrogen (Figs. 3a–d and 4a–e). Trend analysis showed that the most upregulated genes in response to nitrogen were involved in metabolic pathways, biosynthesis of secondary metabolites, and carotenoid biosynthesis (Fig. 8a–f). More nitrogen application upregulated the genes involved in these key pathways, resulting in increased pigment content and dry matter of mutant leaves. Notably, the dry matter of SG mutant leaves with three times more nitrogen was similar to the WT but showed a remarkable increase in the NO_3_-N contents (Fig. 4j), which is due to the lower nitrogen use efficiency in Chl-deficient tobacco plants. Therefore, further study is needed to improve the nitrogen use efficiency (NUE) in Chl-deficient plants.

Currently, the WGCNA method can specifically identify genes related to target traits and obtain co-expression modules with high biological significance [32]. Further, WGCNA is used to pinpoint key regulatory genes, such as hub genes [33]. In this study, seven transcription factor genes and two key genes were identified by WGCNA analysis, and they were directly related to the chlorophyll and NO_3_-N content. The differential roles of these genes have been reported in a few published studies. Xue et al. [34] found that 21 TFs of the 17 families (e.g., bHLH, C3H, MYB, ERF, NAC, and C2H2 families) were co-expressed with the chlorophyll metabolism-related DEGs (e.g., *CLH*) in the L. *japonica* flowers. In *Arabidopsis*, both the *TGA1* and *TGA4* regulate not only the biosynthesis of SA and pipecolic acid (PA) [35] but also the root response to nitrate [36]. *NAC056*, a nuclear-localized transcription activator, plays a critical role in promoting nitrate assimilation and root growth in *Arabidopsis* [37].*OsMYB102* has been reported to perform an important role in directly activating the expression of *OsCYP707A6*, encoding an ABA catabolic enzyme, and thus delaying leaf yellowing [38]. Therefore, we speculate that the transcription factors screened by transcriptome sequencing may result in pigment and nitrate differentiation. Most importantly, we believe that our current research findings deliver valuable bio-information on the candidate pathways and associated genes involved in the determination of tobacco leaf color and NO_3_-N content. However, the function of these identified genes and TFs still needs to be verified. In the future, molecular studies of the genes indentified in this work can be potential targets to dicipher regulatory mechanism of chlorophyll and nitrogen metabolism.

## Conclusion

In conclusion, chloroplast structure had a direct effect on chlorophyll content. Similar biomass, higher photosynthesis, and carbon and nitrogen enzyme activities were achieved when the slight-green mutant was administered three-fold more nitrogen fertilizer, but a higher nitrate content was observed than the wild type seedlings. Transcriptome results revealed that the underlying contributing factor for pigment differentiation in tobacco leaves was the differential gene expression of plant hormone signal transduction, nitrogen metabolism, porphyrin metabolism, and transcriptional regulation. Lower expression level of genes involved in these pathways leads to reduced pigment, which in turn affects related protein synthesis and enzyme activity, resulting in the accumulation of nitrate.

## Materials and methods

### Plant materials and pot experiment setup

The albino and slight-green leaf mutants, isolated from flue-cured tobacco“Zhongyan 100”, were from the Tobacco Research Institute of CAAS (Chinese Academy of Agricultural Sciences). The collection was based on the International Standard for Sustainable Wild Collection of Medicinal and Aromatic Plants (ISSC-MAP) (Version 1.0) prepared by the Medicinal Plant Specialist Group of the IUCN Species Survival Commission (The World Conservation Union). The permission to collect wild plants“Zhongyan 100”from public natural resources is was kindly obtained from the Xuchang Tobacco Research Institute, Xuchang, China. All the study activities complied with the local and national legislation.

Seeds were sown and pot-cultivated at 28 °C under a 12 h/12 h (light/dark) period with a light intensity of 400 µmol m^− 2^ s^− 1^ in a greenhouse located at the National Tobacco Cultivation & Physiology & Biochemistry Research Center of Henan Agricultural University (China) until the seedlings had five to six true leaves. All measurements were made when the phenotype difference between mutants and wild-type was evident. In our preliminary tests, slight-green leaf mutants and wild-type seedlings were treated with different nitrogen concentrations (5, 10, 15, 20, and 25, mmol/L, treated for 7 days) in a form of NH_4_NO_3_ to determine the amount of nitrogen application between two types at the same leaf biomass. We found that a similar biomass was achieved when wild-type was at 5 mmol/L and slight-green leaf mutant was at 20 mmol/L (Figure S2). There were five treatments: (1) NL-Al, albino leaf mutant at 5 mmol/L; (2) NL-SG, slight-green leaf mutant at 5 mmol/L; (3) NL-WT, wild-type seedling at 5 mmol/L; (4) NH-Al, albino leaf mutant at 20 mmol/L; (2) NH-SG, slight-green leaf mutant at 20 mmol/L. “NL” and “NH” represents “Nitrogen Low” and “Nitrogen High”, respectively. For each treatment, 15 uniform plants were selected. After the treatment, five tobacco seedlings from each treatment were blended as biological replicates, and 3 replicates of each treatment were included in the study.

### Sampling

In the pot experiment, leaf samples were taken for physiological and transcriptome analyses when the phenotype difference between mutants and wild-type was evident (seven days after treatment). Expanded leaves of 10 plants were promptly frozen at -196 °C to test the transcriptome, enzyme activity, ammonium (NH_4_)-N, pigment, and soluble protein content. The remaining plants were used to determine the photosynthesis rate and Chl fluorescence before being divided into roots, stems, and leaves for chemical analyses. The plant tissues were heated at 105 °C for approximately 15 min, dried to a constant weight at 65 °C, and then passed through a 60-mesh screen. The dried powder was analyzed for NO_3_-N, total soluble sugar, and reducing sugar content in each sample.

### Observation of chloroplast ultrastructure

Fresh leaves from five treatments were cut into 1.0 × 1.0 mm pieces and immediately fixed in 2.5% glutaraldehyde before being rinsed with 0.1 M PBS three times (15 min for each). Then, the samples were fixed in 1% osmic acid·0.1 M PBS for 5 h at room temperature (20 °C), followed by washing with 0.1 M PBS three times (15 min for each). The washed samples were dehydrated with 30%, 50%, 70%, 80%, 90%, 95%, and 100% ethanol and then placed into an embedding medium. The samples were then sectioned with a Leica UC7 ultramicrotome (Leica Microsystems GmbH, Wetzlar, Germany) and treated with uranyl acetate, followed by lead citrate. Finally, ultrastructural observations were performed by an electron microscope (HT7700, Hitachi High-Technologies Corporation, Tokyo, Japan).

### Measurement of pigment content, net photosynthesis rate (Pn), and enzyme activity

The pigment content was determined using the methods described by Zou [39]. Sucrose synthetase activity (SSA), sucrose phosphate synthase (SPS), rubisco enzyme activity, nitrate reductase activity (NRA), glutamine synthetase activity (GSA), and glutamate synthase (GOGAT) activity were determined using micro determination kits (Suzhou Comin Biotechnology Co., Ltd, Jiangsu, China). The net photosynthesis rate was measured using a portable photosynthetic system (6400XT, LI-COR Biotechnology, Lincoln, NE, USA) 1 h after lights were turned on. The upper third expanded leaves per treatment were selected to measure chlorophyll fluorescence using a PAM-2100 portable Chl fluorometer (Walz, http://www.walz.com). The leaves were first adapted to darkness for 30 min and then illuminated. Then, the actual photochemical efficiency of PSII (Y(II)), PSII maximum photochemical efficiency (Fv/Fm), and potential photochemical activity (Fv/Fo) were measured.

### Measurement of carbonitride content

The NO_3_-N content was determined using the method described by Cataldo et al. [40]. The soluble protein content and NH_4_-N content were determined using micro determination kits (Suzhou Comin Biotechnology Co., Ltd, Jiangsu, China). Total soluble sugar and reducing sugar content were determined according to methods from the Chinese Tobacco Industry standard (YC/T 161, 159–2002).

### RNA extraction, preparation of cDNA libraries, and sequencing

The leaves of plants in the different treatments were used for total RNA isolation using the Trizol reagent kit (Invitrogen, Carlsbad, CA, USA) according to the manufacturer’s protocol. RNA quality was assessed on an Agilent 2100 Bioanalyzer (Agilent Technologies, Palo Alto, CA, USA) and checked using Rnase-free agarose gel electrophoresis. After total RNA was extracted, eukaryotic mRNA was enriched by Oligo (dT) beads. Then, the enriched mRNA was fragmented into short fragments using fragmentation buffer and reverse transcribed into cDNA with random primers. Second-strand cDNA was synthesized by DNA polymerase I, RNase H, dNTP, and buffer. Then the cDNA fragments were purified using a QiaQuick PCR extraction kit (Qiagen, Venlo, The Netherlands), end repaired, a base added, and ligated to Illumina sequencing adapters. The ligation products were size selected by agarose gel electrophoresis, PCR amplified, and sequenced using Illumina Novaseq6000 by Gene Denovo Biotechnology Co. (Guangzhou, China). Quality control of the dataset was performed using fastp (version 0.18.0) [41]. Next, the clean reads were mapped to the K326 tobacco reference genome (https://solgenomics.net/organism/Nicotiana_tabacum/genome) using HISAT2. 2.4 [42] and other parameters were set as default to obtain mapped data.

### Differentially expressed genes (DEGs) selection, gene ontology (GO), and Kyoto encyclopedia of genes and genomes (KEGG) pathway enrichment analysis

The mapped reads of each sample were assembled using StringTie v1.3.1 [43, 44] in a reference-based approach. Expression levels of the genes were calculated using fragments per kilobase of transcript per million mapped reads (FPKM), using RSEM [45] software. RNAs differential expression analysis was performed using DESeq2 [46] software between two different groups. A statistical test was performed using the negative binomial Wald test followed by a Benjamini-Hochberg correction to obtain the false discovery rate (FDR) [46, 47]. DEGs were defined using the following criteria: |log2(FC)| ≥ 2 and FDR < 0.05.

Venn diagrams and heatmaps were generated using OmicShare tools, a free online platform for data analysis (https://www.omicshare.com/tools). All DEGs were mapped to GO terms in the Gene Ontology database [48]. The KEGG pathways were used to assign DEGs with the online KEGG automatic annotation server (KAAS) [49–51]. An FDR below 0.05 was the threshold to identify significant GO terms and enriched pathways in DEGs. Then, the KEGG network was also generated using OmicShare tools.

### Trend analyses of DEGs

To examine the expression pattern of DEGs, the expression data of NL-Al, NL-WT, and NH-Al group (in the order of treatment) and NL-SG, NL-WT, and NH-SG group (in the order of treatment) were normalized to 0, log2 (v1/v0), log2(v2/v0), and then clustered by Short Time-series Expression Miner software (STEM) [52]. The parameters were set as follows: (1) Maximum output profiles number is 20 (similar profiles will be merged); (2) Minimum ratio of the fold change of DEGs is 2.0.

The clustered profiles with an FDR ≤ 0.05 were considered significant profiles. Then, the DEGs in all or each profile were subjected to GO and KEGG pathway enrichment analysis.

### Construction of gene co-expression network

To further explore the regulatory network of gene expression associated with the phenotype of seedlings, gene co-expression networks of 24,882 genes of which FPKM values > 5.8 were constructed using OmicShare tools. The genes were hierarchically clustered based on similarity using the dynamic tree cut algorithm to iform a module with a threshold of minimum module size of 50 genes. The power value used for the analysis was set to β = 8. Additionally, the expression profile of each module was summarized by representing it as the first principal component (referred to as the module eigengene), and the eigengenes of each module were correlated with the parameters to find the key modules associated with chlorophyll and nitrate content. The network file was visualized using Cytoscape (v3.7.1) [53] software to present a core and hub gene biological interaction.

### Gene expression validation using qRT-PCR

Reverse transcription was performed using reverse transcriptase (Vazyme, Nanjing, Jiangsu, China), and then qRT-PCR was performed with SYBR qPCR Master Mix (Vazyme, Nanjing, Jiangsu, China). Reactions were performed in an ABI StepOnePlus Real-Time PCR System (Applied Biosystems, Massachusetts, America), following the manufacturer’s instructions. Three biological replicates were carried out for each treatment, and the primers used for examining the expression of genes are given in Table S4.

### Statistical analyses

Regarding the chemical and physiological characteristics of tobacco plants in the different treatments, correlation analysis and variance between treatments were assessed using SPSS 20.0 (IBM, Palo Alto, CA, USA). Treatments were compared using the least significant difference multiple range test. All data correspond to the mean of three biological replicates (n = 3). Figures were obtained using GraphPad Prism (v. 8.0.1, GraphPad Software Inc., San Diego, CA, USA).

## Electronic supplementary material

Below is the link to the electronic supplementary material.

Supplementary Material 1

Supplementary Material 2

Supplementary Material 3

Supplementary Material 4

Supplementary Material 5

Supplementary Material 6

## Data Availability

The raw sequence data reported in this paper have been deposited in the Genome Sequence Archive [54] in the National Genomics Data Center [55], China National Center for Bioinformation / Beijing Institute of Genomics, Chinese Academy of Sciences (GSA: CRA008504) that are publicly accessible at https://ngdc.cncb.ac.cn/gsa.
