# Supplementary material for: Physiological, transcriptome and co-expression network analysis of chlorophyll-deficient mutants in flue-cured tobacco
Source: BMC Plant Biol. 2023 Mar 22;23:153. doi: 10.1186/s12870-023-04169-z (PMC10031990; doi:10.1186/s12870-023-04169-z)
Supplement: Supplementary file 1 — Supplementary Material 1 [file 12870_2023_4169_MOESM1_ESM.docx]

**Table S1. Data on transcriptome sequencing**

| **Sample name** | **Total reads** | **Mapped reads** | **Mapped ratio (%)** | **Q30 ratio (%)** | **Q20 ratio (%)** | **GC content (%)** | **Exon ratio (%)** |
| --- | --- | --- | --- | --- | --- | --- | --- |
| NL-Al-2 | 45,439,382 | 43,109,488 | 94.87 | 92.71 | 97.27 | 43.89 | 83.40 |
| NL-Al-3 | 36,382,224 | 34,529,782 | 94.91 | 93.27 | 97.46 | 44.01 | 82.95 |
| NL-Al-1 | 41,991,044 | 39,429,152 | 93.90 | 92.38 | 97.05 | 44.06 | 81.85 |
| NL-SG-1 | 36,229,322 | 34,155,521 | 94.28 | 92.80 | 97.27 | 43.80 | 82.56 |
| NL-SG-2 | 46,828,534 | 44,117,202 | 94.21 | 92.24 | 97.03 | 43.82 | 81.72 |
| NL-SG-3 | 37,062,602 | 34,931,924 | 94.25 | 92.64 | 97.12 | 43.97 | 80.40 |
| NL-WT-1 | 35,981,186 | 33,227,804 | 92.35 | 90.86 | 96.25 | 43.98 | 79.67 |
| NL-WT-2 | 40,109,128 | 37,394,081 | 93.23 | 91.56 | 96.24 | 44.55 | 77.53 |
| NL-WT-3 | 43,242,480 | 40,563,941 | 93.81 | 92.40 | 97.10 | 43.68 | 79.32 |
| NH-Al-1 | 42,028,334 | 39,774,871 | 94.64 | 92.81 | 97.49 | 44.38 | 83.66 |
| NH-Al-2 | 35,459,522 | 33,608,290 | 94.78 | 93.57 | 97.81 | 44.30 | 83.70 |
| NH-Al-3 | 38,239,896 | 36,249,170 | 94.79 | 93.07 | 97.60 | 44.16 | 84.07 |
| NH-SG-1 | 46,452,938 | 43,898,176 | 94.50 | 92.95 | 97.56 | 43.85 | 82.27 |
| NH-SG-2 | 48,330,326 | 45,610,763 | 94.37 | 93.01 | 97.59 | 43.70 | 82.82 |
| NH-SG-3 | 46,640,832 | 43,802,155 | 93.91 | 92.94 | 97.56 | 43.85 | 80.97 |
