# Supplementary material for: Physiological, transcriptome and co-expression network analysis of chlorophyll-deficient mutants in flue-cured tobacco
Source: BMC Plant Biol. 2023 Mar 22;23:153. doi: 10.1186/s12870-023-04169-z (PMC10031990; doi:10.1186/s12870-023-04169-z)
Supplement: Supplementary file 4 — Supplementary Material 4 [file 12870_2023_4169_MOESM4_ESM.docx]

**Table S4. Primers used in qRT-PCR.**

| **Primer name** | **Primer sequence (5’–3’)** | **Product length (bp)** |
| --- | --- | --- |
| gene_2672-nF | TCTTTCGGGCTTGCTCCTC | 430 |
| gene_2672-nR | GCTTGCTCTCCAACCTCTTTCC |  |
| gene_23220-nF | TTCGGTTCGTCAACTCTGGT | 267 |
| gene_23220-nR | CTTCGCCTTTGTGCTCCTCA |  |
| gene_42278-nF | AGGTTGTGGTGATTGTGGGT | 262 |
| gene_42278-nR | GGTTTGGTATGGCTGTGTGG |  |
| gene_55784-nF | TCGCTCAATCCACGAACTCT | 418 |
| gene_55784-nR | GTCCAAATACCTTTTCCCCAG |  |
| gene_28134-nF | AGCGTCAGTGGCTATGTATGC | 167 |
| gene_28134-nR | TTGGAGACGCAAGAAAGAGAT |  |
| gene_3180-nF | CCGTCTCAAAAGAACTGGGA | 316 |
| gene_3180-nR | TCCGATAGCAACTCCAAAAAGA |  |
