# Supplementary material for: Physiological, transcriptome and co-expression network analysis of chlorophyll-deficient mutants in flue-cured tobacco
Source: BMC Plant Biol. 2023 Mar 22;23:153. doi: 10.1186/s12870-023-04169-z (PMC10031990; doi:10.1186/s12870-023-04169-z)
Supplement: Supplementary file 5 — Supplementary Material 5 [file 12870_2023_4169_MOESM5_ESM.pdf]

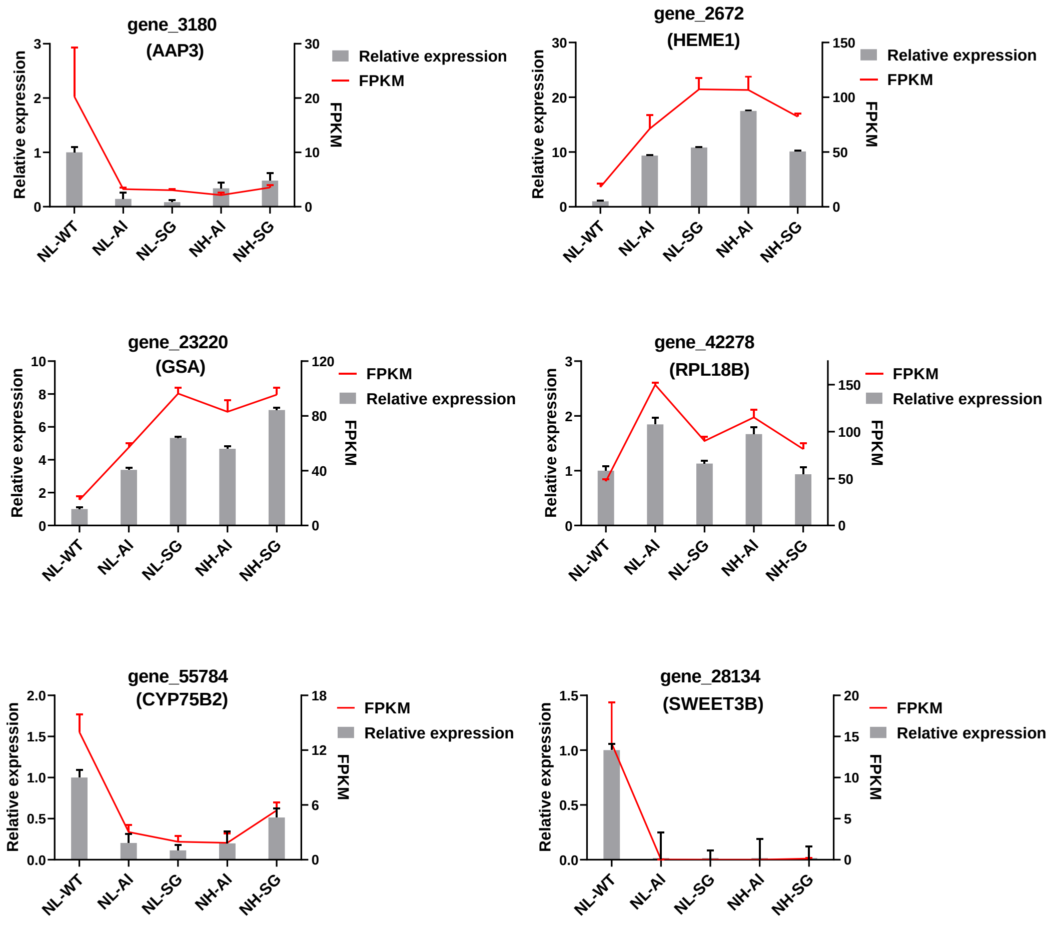


**Figure S1.** RT-qPCR validation of differentially expressed genes. NL-Al, albino leaf mutant at 5 mmol/L; NL-SG, slight-green leaf mutant at 5 mmol/L; NL-WT, wild-type seedling at 5 mmol/L; NH-Al, albino leaf mutant at 20 mmol/L; NH-SG, slight-green leaf mutant at 20 mmol/L. "NL" and "NH" represents "Nitrogen Low" and "Nitrogen High", respectively. The left Y axis represent relative expression and the right Y axis represent FPKM.
