# Supplementary material for: Physiological, transcriptome and co-expression network analysis of chlorophyll-deficient mutants in flue-cured tobacco
Source: BMC Plant Biol. 2023 Mar 22;23:153. doi: 10.1186/s12870-023-04169-z (PMC10031990; doi:10.1186/s12870-023-04169-z)
Supplement: Supplementary file 6 — Supplementary Material 6 [file 12870_2023_4169_MOESM6_ESM.pdf]

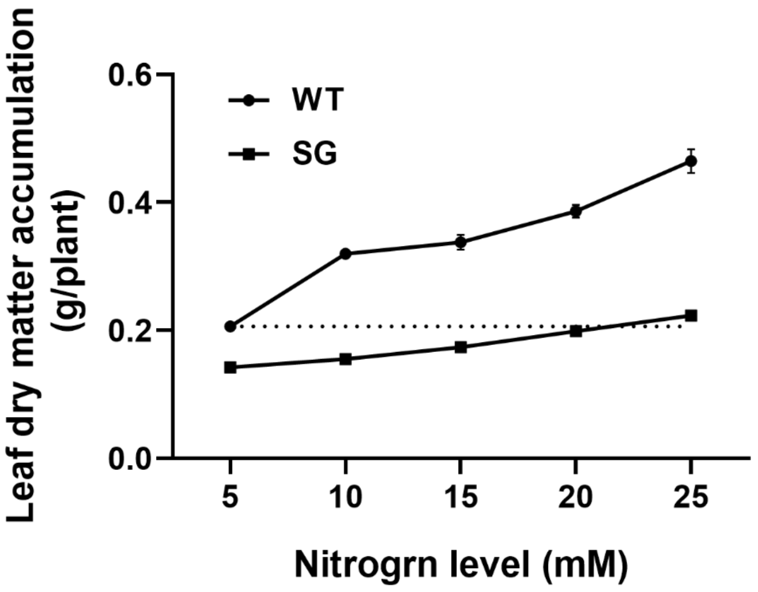

Figure S2. Effect of different nitrogen level on leaf biomass in tobacco. SG, slight-green leaf mutant; WT, wild-type seedling;
